# Comparing endoscopic ultrasonography and double contrast-enhanced ultrasonography in the preoperative diagnosis of gastric stromal tumor

**DOI:** 10.1186/s40644-023-00646-8

**Published:** 2023-12-15

**Authors:** Huiliao He, Tingting Tang, Xiaohua Wang, Lingling Zhou, Liang Wang

**Affiliations:** 1 Department of Ultrasound, The Second Affiliated Hospital and Yuying Children’s Hospital of Wenzhou Medical University, No. 109 West Xueyuan Road, Wenzhou, Zhejiang 325027 China; 2 Department of Pathology, The Second Affiliated Hospital and Yuying Children’s Hospital of Wenzhou Medical University, No. 109 West Xueyuan Road, Wenzhou, Zhejiang 325027 China

**Keywords:** Ultrasonography, Endoscopic Ultrasonography, Gastrointestinal stromal tumors, Diagnosis

## Abstract

**Background:**

This study was designed to perform a comparative analysis between endoscopic ultrasonography (EUS) and double contrast-enhanced ultrasonography (DCEUS) for the preoperative diagnosis of gastric stromal tumors (GSTs).

**Methods:**

A retrospective study was conducted involving 139 patients with histologically confirmed GSTs. All patients preoperatively underwent DCEUS and EUS. The pathology reports were treated as the baseline and were retrospectively compared with the findings of EUS and DCEUS.

**Results:**

Of the 139 lesions, 120 and 113 were correctly identified by DCEUS and EUS, respectively, with an accuracy of 86.3% and 81.3%. The results revealed an insignificant difference between these two methods (*p* = 0.189).

**Conclusions:**

DCEUS can display not only the locations, sizes, shapes, borders, internal echoes, but also show the blood perfusion patterns of GSTs. It is a highly accurate, noninvasive, and convenient method to be used at the pre-treatment stage.

## Background

The annual incidence rate of gastrointestinal stromal tumors (GISTs), possibly malignant mesenchymal tumors occurring in the digestive tract, is approximately 14 to 20 per million [1, 2]. GISTs represent approximately 1 ~ 3% of all primary gastrointestinal malignancies [3, 4]. Gastric stromal tumors (GSTs) comprise 50–70% of GISTs [5, 6]. With the advancements in diagnostic technologies, more GSTs have been found incidentally during routine examinations in recent years. It has been reported that 11–47% of GSTs have distant organ metastases at the time of initial diagnosis [7]. According to the guidelines of the National Comprehensive Cancer Network, GSTs larger than 2 cm should be resected. GSTs less than 2 cm should either be resected or monitored [8]. Therefore, it is necessary to distinguish GSTs from other gastric neoplasms before planning the therapeutic strategies.

Endoscopic ultrasonography (EUS) is used to visualize and generate detailed images of the gastrointestinal tract and surrounding organs [9, 10]. EUS is the preferred mode of imaging for modality in the preoperative diagnosis of GSTs compared to other methods. However, it is invasive and often causes patient discomfort [11], which hampers its application. Thus, a novel method, Double contrast-enhanced ultrasonography (DCEUS), was designed to detect gastric neoplasms in China [12–15]. It combines the oral contrast-enhanced ultrasound with the intravenous microbubbles to obtain more valuable diagnostic information than routine trans-abdominal ultrasonography.

However, there are no published reports comparing the accuracy of EUS and DCEUS in the diagnosis of GSTs. Thus, we performed a retrospective cohort study to explore the benefits of DCEUS in the preoperative diagnosis of GSTs by comparing these two methods.

## Materials and methods

The experimental procedures were sanctioned by the Research Ethics Committee of our Hospital and followed the principles of the Declaration of Helsinki. For this retrospective study, the requirement for informed consent was waived.

### Patients

Four hundred and eighty-six patients were diagnosed with GST in our Hospital between September 2006 and December 2022. The patients who fulfilled the following inclusion criteria were recruited for the study: (a) patients who had been assessed by both EUS and DCEUS; (b) patients whose diagnosis was confirmed by surgery. The exclusion criteria were as follows: (a) patients who had previously received either radiotherapy, chemotherapy, or immunotherapy, (b) patients with contraindications for surgery. There were 139 patients (73 female, 66 male subjects) in the final study group having a mean age of 52.8 ± 9.8 years (range 28–69).

### DCEUS technique

The use of DCEUS with intravenous contrast injection and intraluminal contrast was approved by the Research Ethics Committee of our Hospital before patient enrollment and data collection. DCEUS examinations were performed with Acuson Sequoia 512 or Resona 9T scanner. The ultrasonic oral contrast agent (UOCA) Xinzhang^®^ and the intravenous contrast agent SonoVue were composed of a soya derivative (48 g/package) and sulfur hexafluoride microbubbles (injection), respectively. Intraluminal contrast delineated GST size, shape and margins, while intravenous microbubble contrast agent enabled analysis of tumor vascularity and perfusion patterns. The use of combined intraluminal and intravenous contrast allowed comprehensive evaluation of both tumor morphology and vascularity dynamics.

Under fasting conditions (> 8 h), the patients received 0.5 mg atropine intramuscularly to minimize the peristalsis of the stomach, 30 min before the scan. A multifrequency 4V1 convex array probe was used to perform ultrasonography of the stomach and other abdominal organs to detect baseline tumor morphology and metastatic lesions, respectively. After ingesting 500 mL of UOCA, the patients were assessed in several different positions, and the size, shape, and echoic features of the tumors were recorded, and other gastric lesions were assessed. Next, 2.4 mL of Sonovue was administered as a bolus injection, followed by a 3–5 mL saline flush and DCEUS scanning in the CPS mode. The settings were as follows: acoustic power: -15 to -21 dB; transmission frequency: 1.5 MHz; frame rate, 17–20. We selected a low mechanical index (< 0.2) to prevent microbubble disruption. The enhancement patterns of the gastric lesions in the arterial, venous, and late phases were recorded up to 5 min on tapes. Two experienced, independent, off-site sonographers who were blinded to the study data analyzed these images. On oral contrast-enhanced ultrasonography, GSTs had a round, oval, or lobulated shape and showed a homogeneous hypoechoic or heterogeneous hypoechoic pattern, often due to necrosis from their large size, as well as calcifications within the lesions. Following intravenous contrast administration, the majority of GSTs displayed peripheral ring-like hyperenhancement. Compared to the surrounding normal tissue, GSTs had a significantly higher peak intensity on DCEUS [15].

### EUS technique

An EndoEcho system (host model: EU-M2000) having an Olympus GF-UM 2000-ring with a tip diameter: 12.7 mm, pipe pliers diameter: 2.2 mm, scan range: 360°; Olympus UM-DP12-25R, and UM-DP20-25R ultrasonic microprobe; ultrasonic probe drive MAJ-935; MH-303 bladders.

In a fasted state (> 8 h), the patients were examined from the duodenum to the esophagus (through the lower part of the stomach (antrum, pylorus) to the upper part (gastric body, fundus, cardia)) by inserting the Olympus GF-UM2000 EUS scan-ring into the duodenum. Additionally, the organs surrounding the digestive tract, including spleen, pancreas, retroperitoneum around the aorta, mediastinum, and partial liver, were also examined. Serial images were obtained after advancing the echoendoscope beyond the tumor and pulling back. Deaerated water was used to fill an inflatable balloon around the transducer, which improved the imaging window and increased surface contact. We examined the sizes of the lesions, depths, borders, and the surrounding organs. Two experienced, independent, off-site physicians who were blinded to the study data analyzed the endoscopic images of the target lesions. GSTs found in the stomach through EUS were typically seen in the fourth hypoechoic layer, which corresponds to the muscularis propria, or less commonly in the second hypoechoic layer, the muscularis mucosae. On EUS, they had a hypoechoic appearance and tended to be relatively homogeneous and well-circumscribed. Features that raise suspicion for malignancy included large size (such as > 4 cm, although this cutoff is somewhat arbitrary), irregular borders, lobulations, anechoic spaces within the mass, or hyperechoic foci [16].

### Statistical analysis

The pathology reports were treated as the baseline and were retrospectively compared with the findings of EUS and DCEUS. The data were analyzed using SPSS v22.0. McNemar´s test was used to assess the changes in the rate of diagnostic accuracy between DCEUS and EUS. A *p*-value < 0.05 denoted a statistically significant difference.

## Results

A summary of the general characteristics of 139 cases of GSTs was presented in Table 1. All 139 patients underwent surgery. The resected lesions had diameters in the range of 1.5–13.9 cm (mean 5.5 ± 1.9 cm). Positional distribution of tumors was as follows: 9 cases located in the cardia, 45 cases in the fundus, 41 cases in the body, 16 cases in the angle, 28 cases in the antrum. Based on histological analysis, 62 tumors were classified as low risk, 22 as moderate risk, and 55 as high risk.

**Table 1:** The characteristics of 139 patients with GSTs

	Category	GSTs
Cases, n		139
Gender, male/female		73/66
Age, y, mean ± SD		52.8 ± 9.8
Size, cm, mean ± SD		5.5 ± 1.9
Position, n	Cardia	9
	Fundus	45
	Body	41
	Angle	16
	Antrum	28
Histological classification, n	Low risk	62
	Moderate risk	22
	High risk	55

GSTs, gastric stromal tumors

**Fig. 1:**
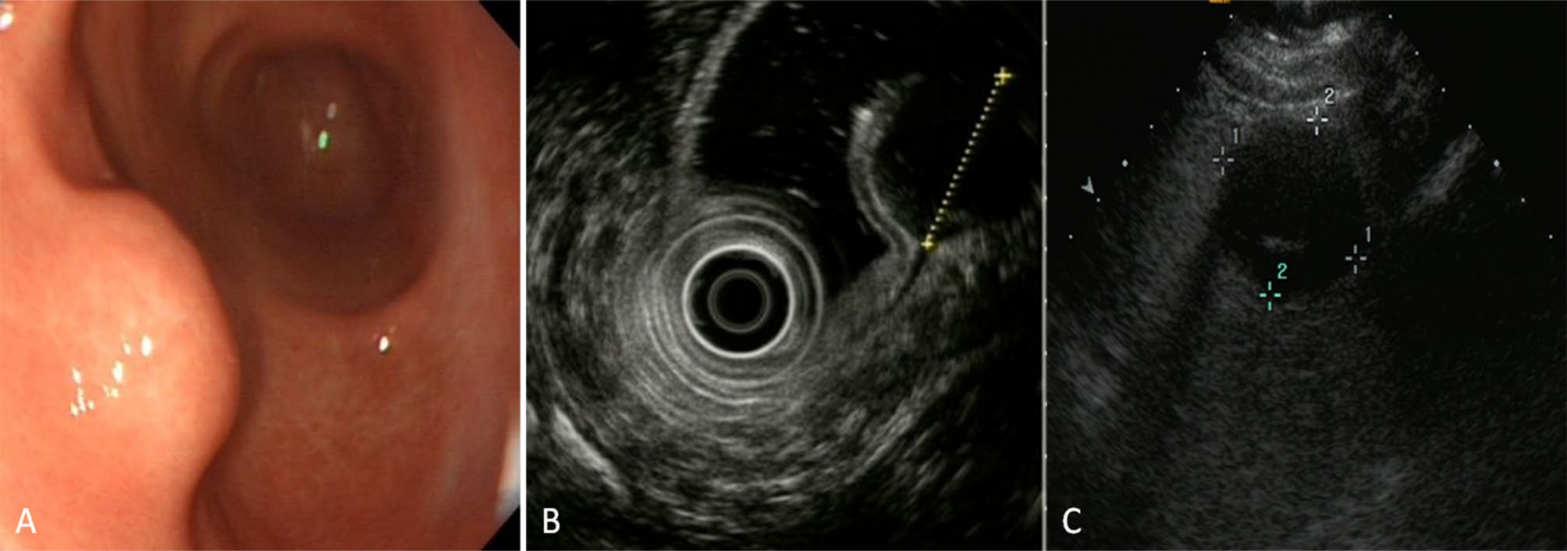
A 53-year-old man with a gastric stromal tumor (GST). (**A**, **B**) Endoscopic ultrasonography (EUS) detected a solid, round, and hypoechoic tumor(calipers) in the gastric antrum. (**C**) Single oral contrast-enhanced ultrasonography (SOCEUS) showed a round, hypoechoic, and heterogeneous mass (calipers) located in the antrum of the stomach

**Fig. 2:**
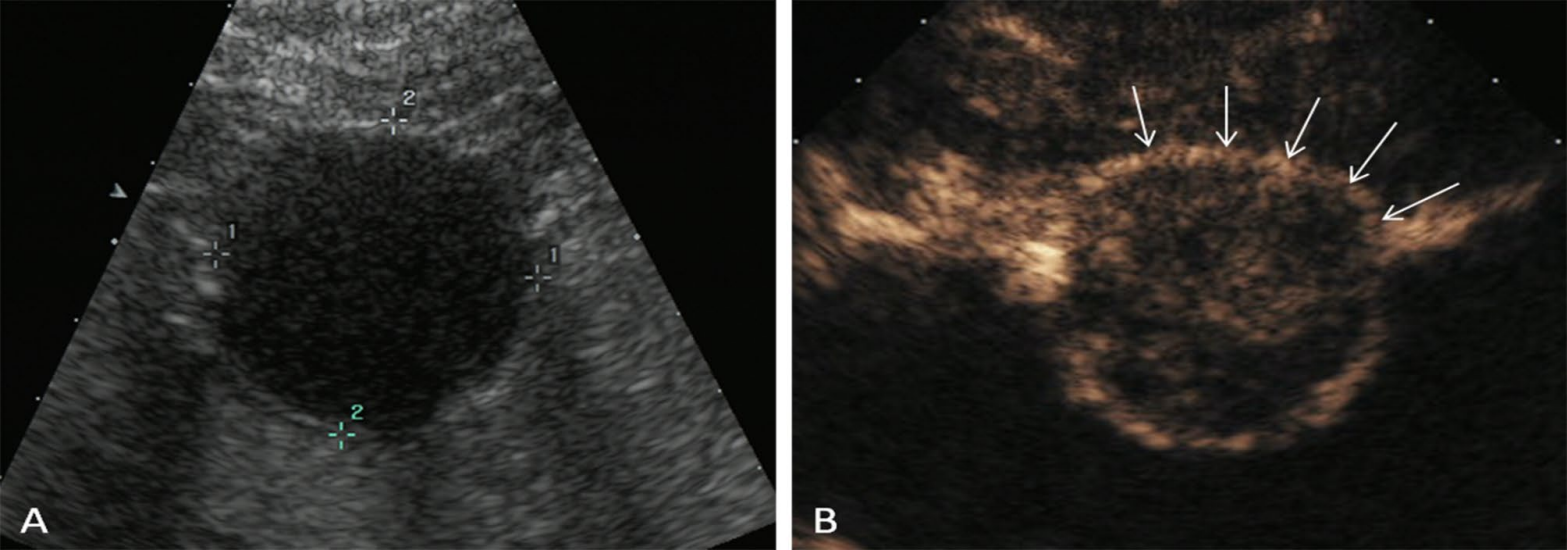
A 49-year-old woman with a GST. (**A**) SOCEUS exhibited a round, hypoechoic, and homogeneous tumor (calipers) in the gastric body. (**B**) DCEUS showed a peripheral ring-like hyperenhancement sign on the tumor (arrows)

**Fig. 3:**
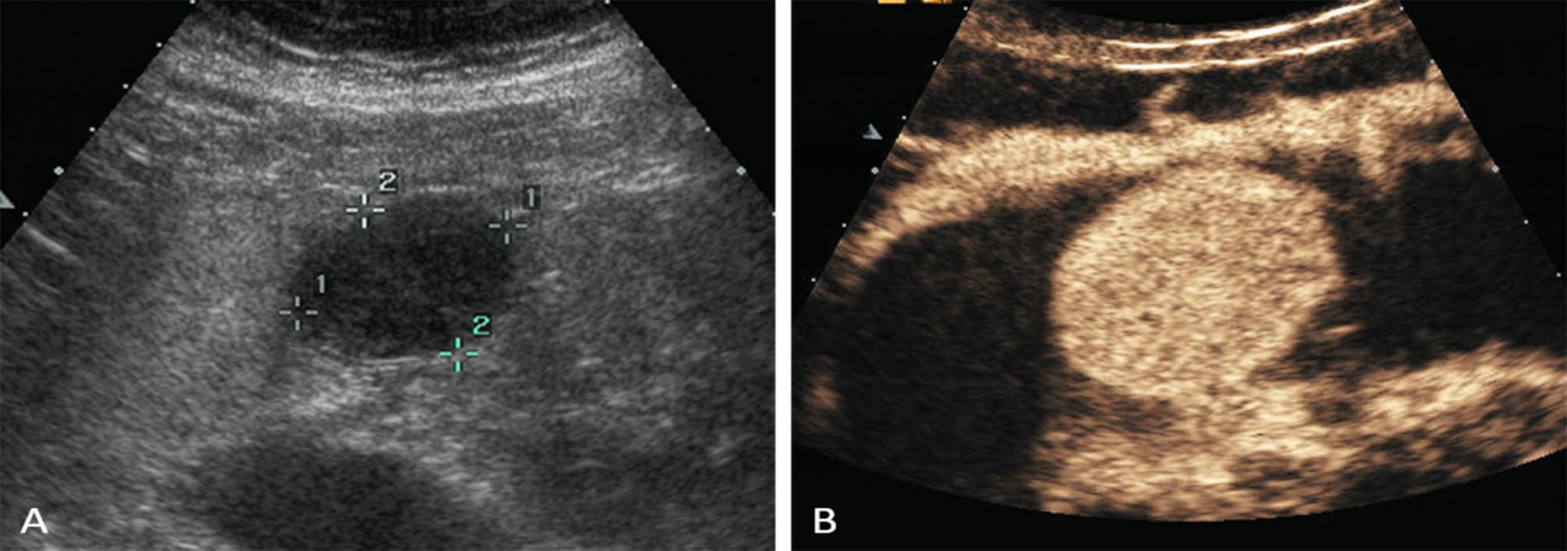
A 46-year-old woman with a GST. (**A**) SOCEUS exhibited an oval-shaped and homogeneous hypoechoic tumor (calipers) in the gastric body. (**B**) DCEUS showed the tumor demonstrated homogeneous enhancement

**Fig. 4:**
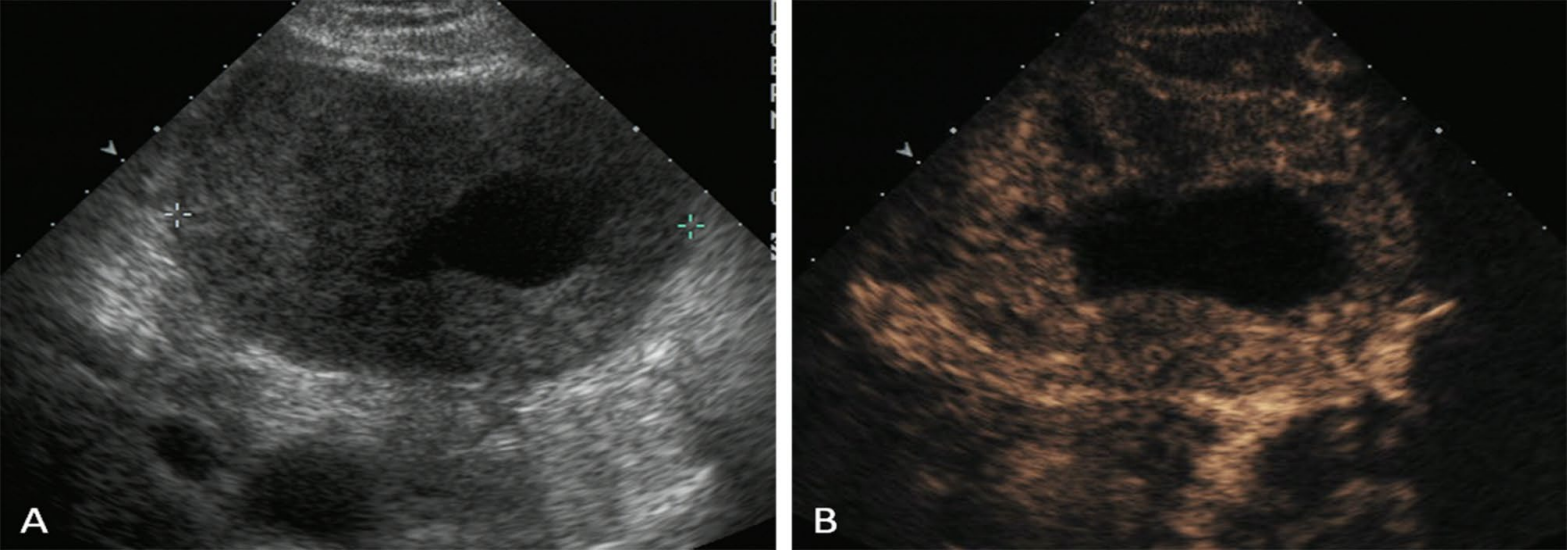
A 60-year-old woman with a GST. (**A**) SOCEUS exhibited a large, ovoid, hypoechoic, and heterogeneous tumor (calipers) in the gastric fundus, cyst formation could be detected in the mass. (**B**) DCEUS showed that the tumor demonstrated heterogeneous enhancement

In both single oral contrast-enhanced ultrasonography and EUS, GSTs performed as round, ovoid, lobulated, dumbbell-shaped hypoechoic masses with internal homogeneous/heterogeneous echogenicity (Fig. 1). Following the bolus injections of Sonovue in DCEUS examinations, the characteristics of GSTs were as follow (Table 2): 78 lesions (56.1%) demonstrated simultaneous enhancement with the normal adjacent gastric wall; 36 lesions (25.9%) exhibited rapid wash-in and fast washout perfusion features; 25 lesions (18%) exhibited rapid wash-in and slow washout perfusion features. Among all 139 GSTs, 115 tumors (82.7%) showed contrast enhancement from the edge to the center and showed a peripheral ring-like hyperenhancement sign (Fig. 2). Eighty-three lesions (59.7%) demonstrated homogeneous enhancement (Fig. 3), and 56 lesions (40.3%) demonstrated a heterogeneous enhancement in the centers of masses (Fig. 4).

**Table 2:** The DCEUS characteristics of 139 lesions

Characteristic	Category	Number (percent)
Perfusion feature	Enhanced synchronously	78(56.1%)
	Fast wash-in and fast wash-out	36(25.9%)
	Fast wash-in and slow wash-out	25(18.0%)
Peripheral ring like hyper-enhancement sign	Present	115(82.7%)
	Absent	24(17.3%)
Central enhancement	Homogeneous	83(59.7%)
	Heterogeneous	56(40.3%)

DCEUS, double contrast-enhanced ultrasonography

Of the 139 lesions, 120 and 113 were correctly identified by DCEUS and EUS, respectively, with an accuracy of 86.3% and 81.3%, respectively. The results revealed an insignificant difference between these two methods (*p* = 0.189) (Table 3).

**Table 3:** Comparison of the two methods in the diagnosis of GSTs

EUS	DCEUS		
	Accurate	Inaccurate	Total
Accurate	106	7	113
Inaccurate	14	12	26
Total	120	19	139

GSTs, gastric stromal tumors; DCEUS, double contrast-enhanced ultrasonography; EUS, endoscopic ultrasonography

P = 0.189

## Discussion

GST has received a lot of attention in recent years due to its unique clinicopathological and biological characteristics [17, 18]. GSTs start at in the muscularis propria of the stomach and are usually located in the fundus [19, 20]. Small GSTs often form either solid intramural or subserosal or rarely intraluminal polypoid masses. Larger GSTs generally occur in the form of external, occasionally pedunculated masses that are attached to the outer muscular layers of the gut. Most larger tumors present as large cysts. Most GSTs remain ‘silent’ until reaching a large size. The symptoms are based on the tumor’s location and size. Nonspecific symptoms, such as fatigue, dyspepsia, anorexia, abdominal pain, nausea, and weight loss, are observed in symptomatic patients. Occasionally, bleeding associated with tumor rupture or mucosal ulceration is also observed. Some patients with large GSTs may have externally palpable masses [21]. The common metastatic sites of aggressive GSTs, including liver and other abdominal organs; however, metastasis generally occurs within 10–15 y post-diagnosis of the primary tumor [22]. Lymph node metastasis is uncommon.

The diagnosis of GSTs is based on imaging techniques. Many imaging modalities can be used to identify GSTs, including endoscopy, computed tomography (CT), positron emission tomography (PET), and magnetic resonance imaging (MRI) [17, 23, 24]. However, CT scan involves ionizing radiation [25]; MRI cannot be performed with pacemakers or cochlear implants [26]; PET scan is very expensive [27]. Also, it is difficult to identify small lesions vis endoscopy in cases of GSTs, which grow typically in the muscularis and subserosa [28].

EUS is a vital technique used in the diagnostic work-up of GSTs. Some researchers studied the value of EUS and found it to be efficient and highly accurate in the preoperative diagnosis of GSTs [29]. However, patient discomfort during examination limits its application, and the origin may be difficult to identify when the mass is typically exophytic and large. DCEUS is a simple, cheap, convenient, noninvasive, and reliable transabdominal ultrasound technique. It enhances sonographic visualization by using both intravenous and intraluminal contrast agents. Here, we found an insignificant difference in the diagnostic accuracy of DCEUS and EUS. DCEUS clearly showed the locations, sizes, shapes, borders, and internal echogenicities of GSTs. Moreover, DCEUS could display blood perfusion patterns of the tumors. In the presence of an oral contrast agent, the gas in the stomach gets exhausted, forming a uniformly distributed sound transmission interface, leading to a depletion in the ultrasonic artifacts and displaying a clear gastric wall; thus, increasing the rate of detection of the lesions of the stomach and the surrounding organs [30]. An intravenous contrast agent, SonoVue, enhances echogenicity, displays real-time blood perfusion in tumors, and increases visualization to identify the margins of lesions [31, 32]. Thus, DCEUS can display both morphology and perfusion characteristics of gastric tumors. GSTs are generally found in the muscularis with intact layers of gastric walls. GSTs receive blood from both mucosa and serosa, forming a perfusion pattern from the edge to the center, which explains the simultaneous enhancement in the lesions and the normal adjacent gastric wall along with a peripheral ring-like hyperenhancement sign that were observed in this study. Internally, the GSTs may exhibit hemorrhage, necrosis, or cyst formation due to the different sizes and degrees of malignancy [33–35], consistent with the DCEUS appearance. Thus, a gastric mass with a peripheral ring-like hyper-enhancement sign and centripetal filling enhancement pattern with or without internal necrosis or cyst formation likely indicates a GST. An important advantage of EUS is the ability to perform fine needle aspiration (FNA) biopsy for cytological analysis during the same procedure. FNA allows definitive tumor characterization and is recommended by treatment guidelines. In contrast, tissue sampling cannot be obtained concurrently with DCEUS. This is a limitation of DCEUS compared to EUS, although DCEUS findings may be able to guide subsequent biopsy.

A major limitation of this study was the modest sample size and the retrospective study design. To definitively establish the diagnostic accuracy of DCEUS for GSTs, large-scale prospective studies are required to minimize potential biases and enhance generalizability of findings. We hope the preliminary findings presented here provide a foundation to inform future prospective research with robust methodology and increased statistical power.

## Conclusion

DCEUS can not only display the locations, sizes, shapes, borders, internal echoes but also show the blood perfusion patterns of GSTs. Its diagnostic accuracy in GSTs is similar to that of EUS. DCEUS is a highly accurate, noninvasive, and convenient method to be used at the pre-treatment stage. Further research through prospective trials with larger sample sizes is warranted to validate the diagnostic utility and accuracy of DCEUS compared to other imaging modalities.

## Data Availability

The analyzed and/or used datasets in this study can be obtained on reasonable request to the corresponding author.

## Abbreviations

GISTs: gastrointestinal stromal tumors
GSTs: gastric stromal tumors
EUS: endoscopic ultrasonography
DCEUS: double contrast-enhanced ultrasonography
CT: computed tomography
PET: positron emission tomography
MRI: magnetic resonance imaging
FNA: fine needle aspiration
